# Optimized Copper-Modified Zinc Oxide Photoanodes for Solar-to-Hydrogen Evolution

**DOI:** 10.1021/acsami.5c17721

**Published:** 2026-01-10

**Authors:** Premrudee Promdet, Fan Cui, Raul Quesada-Cabrera, Sanjayan Sathasivam, Jiang Wu, Claire J. Carmalt, Ivan P. Parkin

**Affiliations:** † Materials Chemistry Centre, Department of Chemistry, UCL (University College London), 20 Gordon Street, London WC1H 0AJ, U.K.; ‡ Department of Electronic & Electrical Engineering, UCL (University College London), Malet Place, London WC1E 7JE, U.K.; § Department of Chemistry, Institute of Environmental Studies and Natural Resources (i-UNAT), 16750 Universidad de Las Palmas de Gran Canaria (ULPGC), Campus de Tafira, Las Palmas 35017, Spain; ∥ School of Engineering & Design, 4914 London South Bank University, 103 Borough Road, London SE1 0AA, U.K.; ⊥ Energy, Materials & Environment Research Centre, London South Bank University, 103 Borough Road, London SE1 0AA, U.K.; # Institute of Fundamental and Frontier Sciences, 12599 University of Electronic Science and Technology of China, Chengdu 610054, P. R. China

**Keywords:** photoelectrochemical hydrogen evolution, copper-modified zinc oxide, plasmonic photocatalysts, cost-efficient photoanodes, chemical vapor deposition

## Abstract

This work presents a single-step method for producing cost-efficient copper-modified zinc oxide photoanodes through scalable chemical vapor deposition. The role of Cu incorporation is thoroughly investigated, with the identification of an optimized loading of the metal in these films. The optimally Cu-modified ZnO sample (CZO-5.6) achieved a stable photocurrent of approximately 1.22 mA cm^–2^ at 1.23 *V*_RHE_, along with a Faradaic efficiency of 89%. This enhanced performance was attributed to surface plasmon resonance (SPR) effects induced by copper nanoparticles, as evidenced by photoluminescence spectroscopy results. To promote stability under the experimental conditions of the PEC cell, the best-performing photoanode was further protected using amorphous TiO_2_ deposited by atomic layer deposition. Amorphous TiO_2_ coatings have been found to be exceptionally stable in alkaline solutions and highly conductive for photogenerated holes, offering a promising solution for PEC electrode protection. This work not only describes a method for fabricating photoanodes with high photocatalytic activity but also suggests a low-cost route toward the development of photocatalysts for hydrogen production.

## Graphical abstract

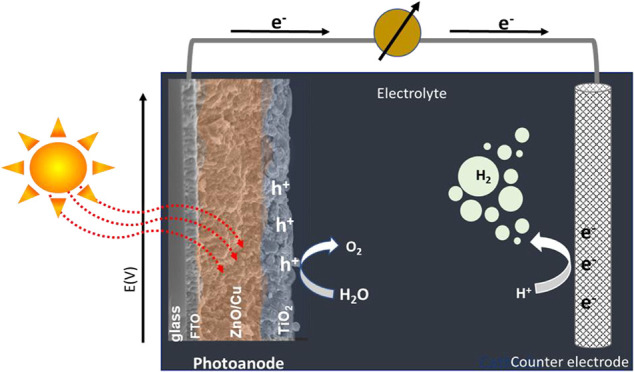

## Introduction

Photoelectrochemical (PEC) water splitting is a promising route to sustainable hydrogen production, offering a direct method to convert solar energy into chemical fuel.^1^ In a typical PEC cell, a photoanode (i.e., a light-absorbing semiconductor electrode) is immersed in an electrolyte and illuminated to drive the oxygen evolution reaction (OER).^2^ This is paired with a counter electrode, typically platinum, which catalyzes the hydrogen evolution reaction (HER). While the HER is well understood and efficiently catalyzed under most conditions, overall water splitting is limited by the sluggish kinetics of the OER. An efficient HER can only proceed if the OER occurs at a comparable rate; however, the OER involves complex four-electron transfers and multiple proton-coupled steps, making it inherently more challenging.^1–3^ As such, substantial research has focused on developing photoanode materials that enhance OER performance by improving light absorption, charge separation, and interfacial charge transfer.^3,4^

Various semiconductors, such as TiO_2_ and Fe_2_O_3_, have been explored as photoanodes.^5–7^ Among them, zinc oxide (ZnO) is particularly attractive due to its wide band gap (3.37 eV); suitable valence band position for water oxidation; and advantages in abundance, sustainability, and low cost.^8^ ZnO also exhibits exceptionally high electron mobility (up to 100 times greater than that of TiO_2_), which facilitates effective separation of photogenerated charge carriers, thereby suppressing recombination and enhancing photocurrent generation. Its high surface reactivity further promotes interfacial redox processes.

Despite these advantages, ZnO is photoactive only under UV light, which constitutes only ∼4% of the solar spectrum. It is also prone to photocorrosion in aqueous environments under illumination.^9,10^ These drawbacks have motivated a wide range of material engineering strategies to enhance ZnO’s PEC performance, including doping to tune its electronic structure, heterojunction formation to improve charge separation, and surface modification with plasmonic metals to expand light harvesting into the visible region.

Among these, plasmonic modification has proven to be especially promising. When illuminated, plasmonic metal nanoparticles exhibit surface plasmon resonance (SPR), generating intense local electric fields and energetic hot carriers. In metal/n-type semiconductor systems, hot electrons with sufficient energy can overcome the Schottky barrier at the interface and be injected into the conduction band of the semiconductor, contributing to the photocurrent.^11,12^ Simultaneously, hot holes left behind on the metal or photogenerated holes in the semiconductor drive the OER. SPR-induced effects also enhance light absorption and promote spatial charge separation, both of which are beneficial for PEC performance.^12^

Noble metals such as Au, Ag, and Pt have been widely studied for their SPR effects in PEC systems.^13–16^ However, several earth-abundant non-noble metals also exhibit SPR and can similarly improve the PEC activity. Among these, copper (Cu) is particularly attractive due to its large extinction cross section, photosensitivity, high conductivity, and low cost.^11,17^

Previous studies on Cu–ZnO systems have primarily focused on substitutional Cu doping to form solid solutions or on CuO–ZnO and Cu_2_O–ZnO composites.^18–21^ For systems involving metallic Cu, the typical approach involves a two-step process: ZnO nanostructures are first synthesized via solvothermal methods, followed by Cu deposition using electrodeposition or physical vapor deposition (PVD).^22,23^ These systems have largely been explored for electrocatalysis (e.g., nitrate-to-ammonia conversion) or thermal catalysis (e.g., CO_2_ hydrogenation), rather than for PEC water splitting.^22,23^

To harness the plasmonic properties of Cu nanoparticles (NPs) for PEC applications, this study presents a single-step synthesis of Cu-modified ZnO photoanodes by using aerosol-assisted chemical vapor deposition (AACVD) under a nitrogen atmosphere. This scalable approach enables precise tuning of film properties by adjusting precursor chemistry, solvent, and deposition parameters.^24–28^ The influence of Cu incorporation was systematically investigated, and an optimal Cu loading was identified for maximizing PEC hydrogen production. To improve stability under operational conditions, the best-performing photoanode was coated with amorphous TiO_2_ via atomic layer deposition (ALD). Amorphous TiO_2_ has demonstrated excellent stability in alkaline environments and high hole conductivity, making it an effective protective layer for PEC electrodes.^29,30^

## Experimental Section

All chemical precursors were used as received from Sigma-Aldrich. Cu-modified ZnO films were deposited from copper acetate monohydrate (99%) and zinc acetate dihydrate (98%) in methanol (99.8%) using aerosol-assisted chemical vapor deposition (AACVD). In this system (Scheme S1), an ultrasonic humidifier (Johnson Matthey) created a mist from the precursor mixtures, which was carried into a cold-wall reactor under a nitrogen flow (BOC, 1.0 l min^–1^). The products were deposited onto fluorine-doped tin oxide (FTO) silica-barrier float glass substrates (cleaned using distilled water/detergent mix, isopropyl alcohol, and finally acetone before drying in a 70 °C oven) from NSG Pilkington Ltd. at 400 °C. The resulting films were adherent and passed the conventional Scotch tape test. A series of samples was prepared from precursor solutions containing a fixed concentration of zinc acetate (0.05 M) and varying concentrations of copper acetate (0, 10^–3^, 2·10^–3^, 4·10^–3^, 8·10^–3^ M, respectively) in a total volume of 60 mL. In specific samples, a ∼7 nm TiO_2_ protective layer was deposited from titanium isopropoxide using atomic layer deposition (Ultratech, Savannah G2 S200) at 100 °C and a deposition rate of 0.48 Å per cycle.^31^

Film morphology and thickness were studied using top- and side-view scanning electron microscopy (SEM) on a JEOL6301 instrument operated at 10 kV. X-ray diffraction (XRD) analysis was carried out using a Bruker-AXS D8 (GADDS) diffractometer equipped with a monochromated Cu X-ray source emitting Cu Kα1 (λ = 1.54056 Å) and Cu Kα_2_ (λ = 1.54439 Å) radiation at an intensity ratio of 2:1 and a 2D area X-ray detector with a resolution of 0.01°. Films were analyzed under a glancing incidence angle (θ) of 1°. The diffraction patterns were refined using database standards. UV/vis spectroscopy was performed using a double monochromated PerkinElmer Lambda 950 UV/vis/NIR spectrophotometer in the 300–800 nm range. X-ray photoelectron spectroscopy (XPS) was performed using a Thermo Kα spectrometer with monochromated Al Kα radiation, a dual-beam charge compensation system, and a constant pass energy of 50 eV. Survey scans were collected over the range 0–1200 eV. High-resolution XPS spectra were acquired for the principal peaks of Zn (2p), Cu (2p), and Ti (2p) and fitted using CasaXPS software with the calibration of C 1s at 284.5 eV. Room temperature photoluminescence (Renishaw 1000) spectroscopy was employed to study the optical properties of the ZnO films using a He–Cd laser (λ= 325 nm, *E* = 3.8 eV). The surface roughness was characterized by atomic force microscopy (AFM) on a Keysight 5600LS scanning probe microscope, with a scan size of 5 μm × 5 μm. High-resolution TEM (HR-TEM) was performed using a JEOL 2100 (200 kV) instrument fitted with a LaB6 filament, giving a point resolution of 0.13 nm. The instrument was equipped with bright- and dark-field STEM detectors and an Oxford Instruments EDS detector.

Photoelectrochemical studies were conducted using a 0.5 M Na_2_SO_4_ electrolyte solution (Fluka) at pH 7.0 in a three-electrode electrochemical cell, with Ag/AgCl and Pt serving as the reference and counter electrodes, respectively. The potential was controlled by a potentiostat (Ivium Technology), ranging from −1.0 to 1.23 V vs Ag/AgCl at a scan rate of 50 mV s^–1^. The photocurrent density (*J*) and electric potential (*V*) of the photoanode were measured by linear scanning voltammetry. Measurements were performed in the dark and under chopped and continuous illumination, respectively, using an Ivium Compact Stat lamp (Ivium Technologies). The emission spectrum of the lamp is shown in Figure S1 for reference. The irradiance of the lamp (100 mW cm^–2^, AM 1.5 G) was calibrated by using a silicon reference cell with an optical meter (Newport, Model 1918-R). Stability tests were conducted under AM 1.5 sun illumination in 0.5 M Na_2_SO_4_ at pH 7.0, with a constant potential of 0.6 V vs RHE. Hydrogen generation in the PEC cell was monitored using gas chromatography (Shimadzu GC-2014) under a bias voltage of 0.6 V vs Ag/AgCl. The theoretical H_2_ rate was obtained using eq 1.^32^

1 $$H_{2}=\frac{Q}{2F}=\frac{I\cdot t}{2F}=\frac{1}{2}\frac{\left(\int_{o}^{t}Idt\right)}{F}$$

where *Q* (units, C s) is the charge passing through the circuit during a period of *t* (units, s), *I* is the photocurrent, and *F* is the Faraday constant (96,484.34 C mol^–1^). The *Q* value is estimated by integrating the current over time when the current is not constant. The Faradaic efficiency, FE, was estimated from the ratio between experimental and theoretical H_2_ rates.

## Results and Discussion

A series of Cu-modified ZnO films were deposited in a single step from methanolic mixtures of zinc acetate (0.05 M) and copper acetate (0, 10^–3^, 2·10^–3^, 4·10^–3^, 8·10^–3^ M, respectively) using aerosol-assisted (AA) CVD (Figure S1) at 400 °C under a nitrogen atmosphere. Energy-dispersive X-ray spectroscopy (EDS) analysis showed the Cu content in the films to be 0, 2.2, 3.7, 5.6, and 15.2 at %, respectively, relative to Zn, thus increasing linearly with the increase in copper acetate concentration in the precursor solution. The films are henceforth referred to as CZO-(Cu at%), as listed in Table 1. EDS mapping analysis was also used to verify the uniform distribution of Cu in the samples (Figure S2). Scanning electron microscopy (SEM) and atomic force microscopy (AFM) images showed that the films consisted of spherical particles with diameters ranging from 50 to 100 nm (Figure 1). Clustering of these particles into larger features, several hundred nanometers in width, was also observed across all films, with the effect most pronounced in the CZO-15.2 sample. The consistency in film morphology and surface area across the five CZO films is advantageous, as it allows us to dismiss any significant difference in performance to be due to variations in surface area.

**Figure 1:**
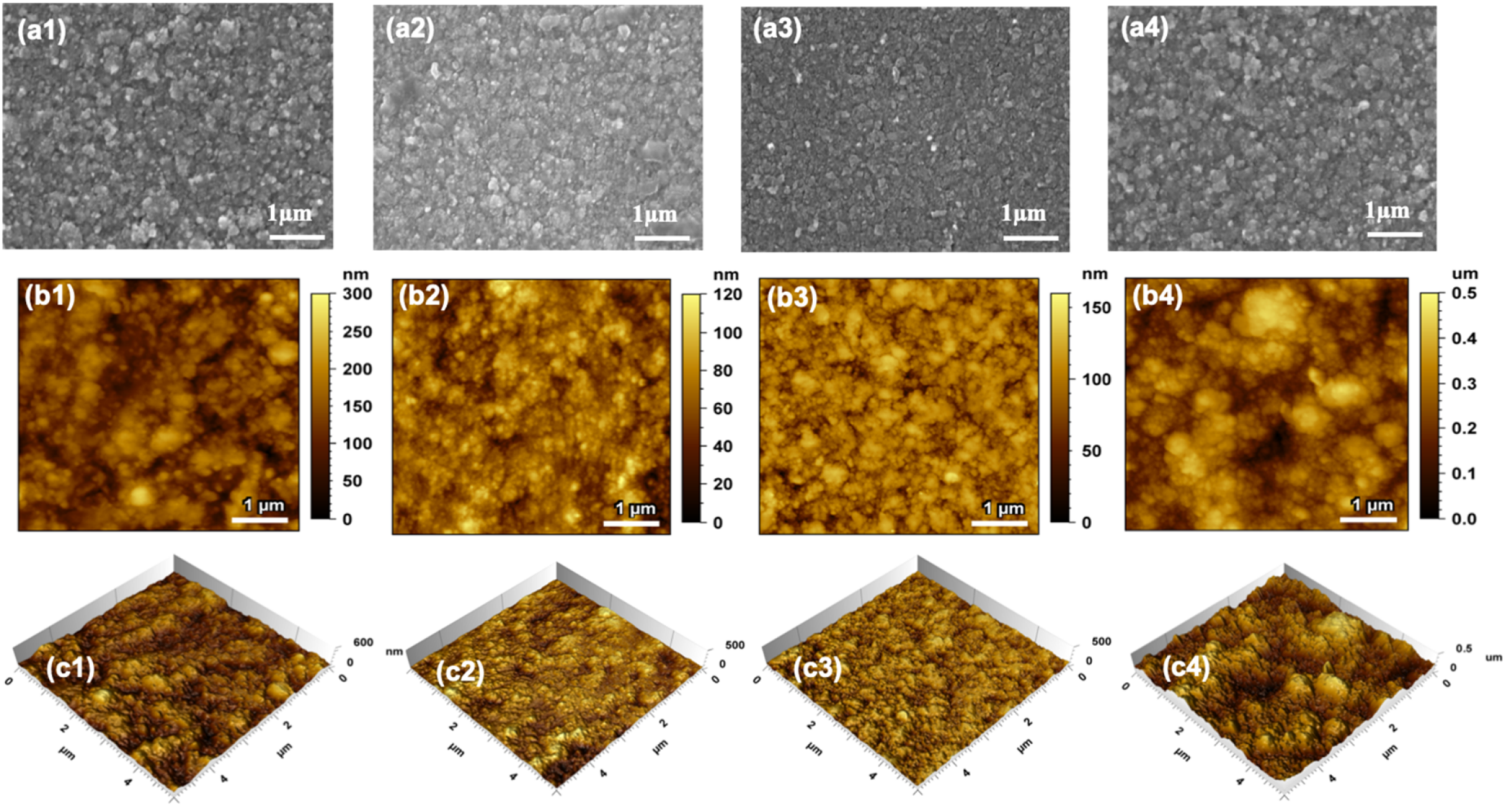
(a) Scanning electron microscopy (SEM) and (b, c) atomic force microscopy (AFM) images of the CZO films with increasing Cu content: (1) 2.2 at%, (2) 3.7 at%, (3) 5.6 at%, and (4) 15.2 at%. (b) and (c) 2D and 3D AFM images, respectively.

**Table 1:** Physical and Functional Parameters of Pure ZnO and CZO Films with Varying Cu Contents (at %)^a^

		lattice parameters					
sample	[Cu]/at %	*a*, *b* (Å)	*c* (Å)	*V* (Å^3^)	thickness/nm	SA/μm^2^	J/mA cm^–2^
CZO-0	0	3.25216	5.21218	47.74	1020 ± 36	25 ± 1	0.32
CZO-2.2	2.2	3.25481	5.21757	47.87	1150 ± 55	29 ± 3	0.55
CZO-3.7	3.7	3.25658	5.20835	47.84	990 ± 80	26 ± 2	0.74
CZO-5.6	5.6	3.25564	5.2164	47.88	1100 ± 20	29 ± 4	1.22
CZO-15.2	15.2	3.25569	5.21012	47.83	1050 ± 15	31 ± 3	0.60
TiO2@CZO-5.6	5.6	3.25564	5.2164	47.88	1150 ± 25	27 ± 2	1.43

a Physical data include lattice parameters and unit cell volume (*V*), film thickness, and surface area (SA). Photocurrent density (*J*) was obtained at 1.23 *V*_RHE_.

High-resolution TEM analysis (Figure 2) also confirmed that the films were composed of particles with diameters up to 100 nm. The images clearly show crystal lattice fringes characteristic of metallic Cu, surrounded by amorphous or highly disordered copper oxide material, and, at a longer range, the crystalline ZnO host. The lattice spacing in the nanoparticle was 0.210 nm, corresponding to the (111) plane of Cu.^33,34^ Interplanar spacing values of 0.271 nm were attributed to the (002) planes of hexagonal ZnO.^35^

**Figure 2:**
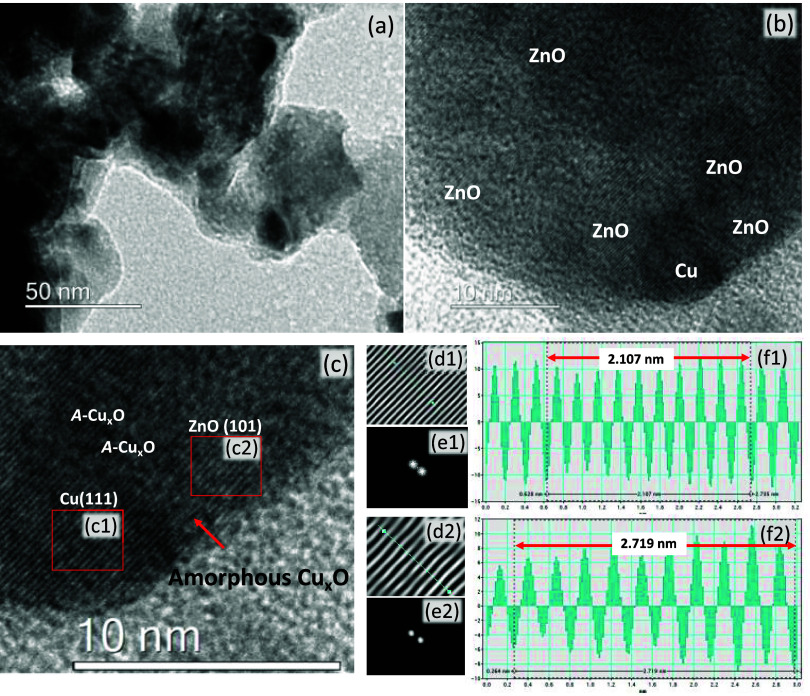
(a–c) High-resolution transmission electron microscopy (HR-TEM) images of sample CZO-5.6, highlighting Cu and ZnO areas and amorphous regions attributed to *a*-CuO, (d) inverse fast Fourier transform (IFFT) transformation images, (e) fast Fourier transform (FFT) reciprocal-lattice image, and (f) corresponding lattice spacing line profile of the IFFT images of regions c1 and c2.

XRD analysis confirmed the formation of hexagonal wurtzite ZnO in all samples, with additional peaks corresponding to cubic metallic Cu appearing in films containing 3.7 atom % Cu and above (Figure 3a). It is important to note that metallic Cu may also be present at lower doping levels but in quantities too small to be detected by XRD. The Le Bail fitting of the data was carried out to assess the impact of Cu doping on ZnO unit cell parameters (Table 1). However, due to the similar ionic radii of Zn­(II) and Cu­(II) (0.74 and 0.73 Å, respectively), no significant structural differences were observed.

**Figure 3:**
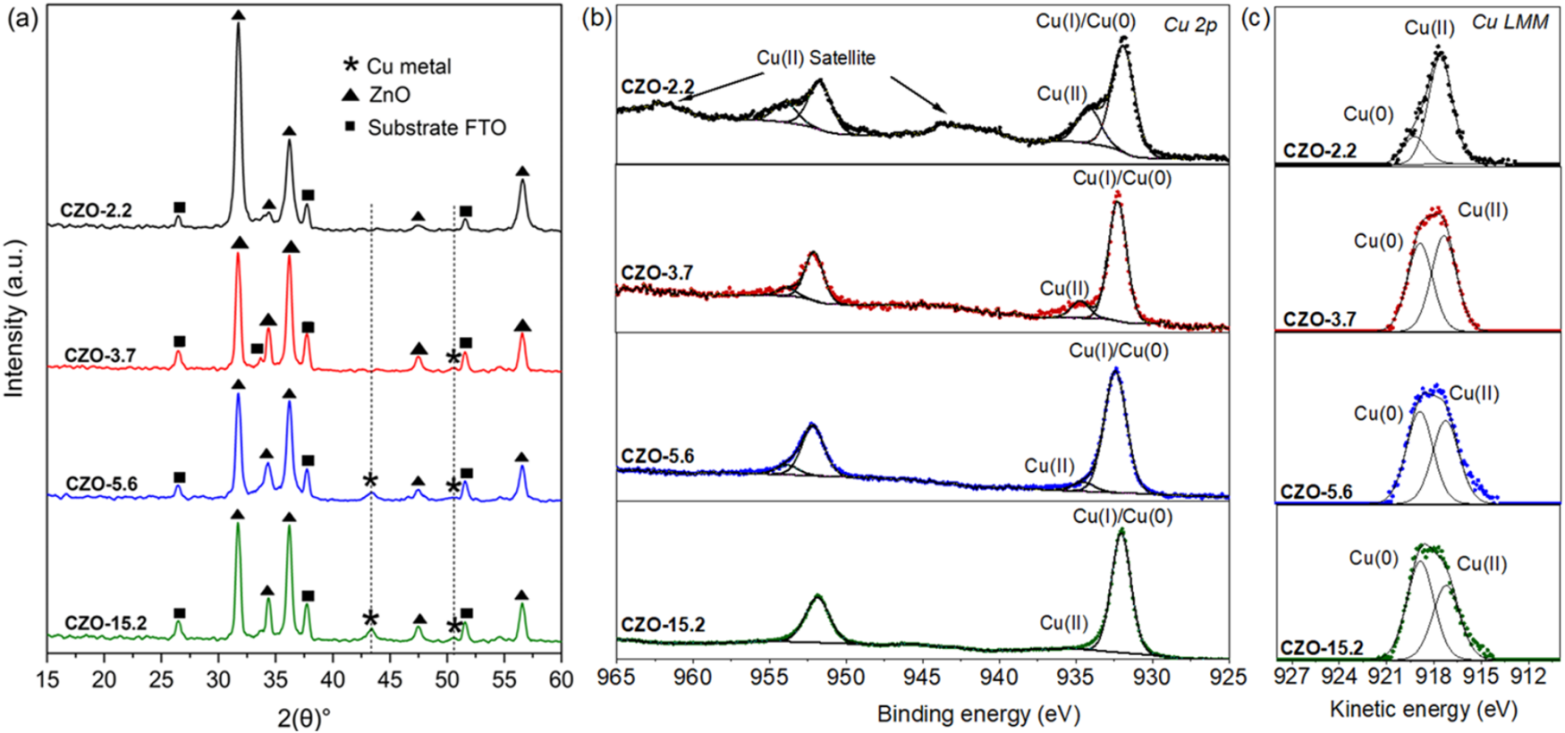
(a) XRD patterns of CZO films with varying Cu contents (2.2, 3.7, 5.6, and 15.2 at%, respectively). Triangle symbols correspond to wurtzite ZnO (JCPDS 00-036-1451), star symbols correspond to cubic Cu metal (JCPDS 65-9026), and square symbols correspond to the FTO substrate. (b) High-resolution XPS binding energies in the Cu 2p region and (c) corresponding Cu LMM spectra.

Further characterization was carried out using XPS (Figure 3b), with the identification of surface Cu(0) metal centers and Cu­(II) ions (2p_3/2_ peaks at 932.6 and 934.7 eV, respectively). It is worth noting that the area of the Cu(0) peak increased relative to that of the Cu­(II) peak upon increasing the Cu content, as it corresponds to the growth of metal nanoparticles on the film surfaces. Likewise, the Cu LMM Auger peak attributed to the kinetic energy of Cu(0) at 918.2 eV increased relative to that of Cu­(*II*) at 917.2 eV upon increasing the Cu content in the films (Figure 3c). The presence of surface Cu­(II) species supports the identification of amorphous/disordered copper oxide (*A*-CuO), as noted in the HR-TEM studies.

The formation of Cu NPs had a notable impact on the optical properties of the films (Figure 4a), with an absorption band emerging at *ca*. 580 nm upon increasing the Cu content. A red shift and broadening of this band in sample CZO-15.2 correlated with the growth of large, aggregated particles and is consistent with previous observations of surface plasmon resonance (SPR) from Cu NPs.^36^

**Figure 4:**
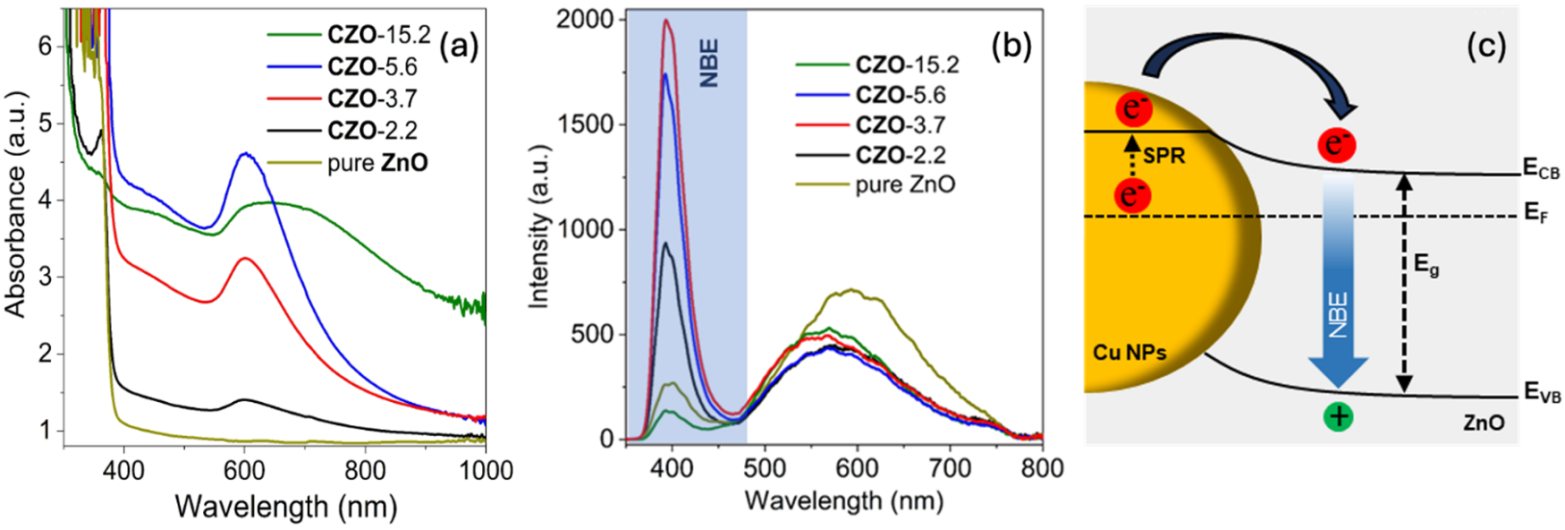
(a) Absorbance spectra of pure ZnO and CZO films, (b) corresponding photoluminescence spectra recorded under an excitation wavelength of 325 nm, and (c) schematic illustration of the energy band diagram of the CZO system showing the enhancement of near-band-edge (NBE) emission from hot electrons generated from the SPR effect.

Photoluminescence (PL) studies showed typical bands in the UV (near-band-edge, NBE) and visible regions (Figure 4b). The NBE of ZnO is usually located within 368–380 nm, which is due to the radiative recombination of free excitons.^37^ In our case, the NBE was red-shifted to ∼390 nm likely due to surface defects, which are commonly observed for ZnO.^38^ Surface defects can induce band bending, effectively reducing the band gap near the surface and contributing to charge recombination at lower energies compared to those in the bulk, hence a red shift of the NBE band.^39^ These recombination centers would also contribute to the quenching of NBE emission, as observed for the nominally pure ZnO in our studies, with a weak NBE band relative to the features in the visible region (Figure 4b). The broad band in the visible region (450–750 nm) is typically assigned to luminescence due to structural defects.^40^ Doping or passivation of surface states associated with deep-level defects (i.e., oxygen vacancies, zinc interstitials, etc.) upon incorporation of Cu can lead to emission from shallow defect levels, inducing a blue shift in the visible emission, as observed in the case of the CZO films (Figure 4b). It is interesting to note that the NBE peak in the CZO films was not shifted relative to that in pure ZnO. The formation of a Schottky barrier upon the deposition of Cu NPs would lead to changes in the local band structure near the surface, which is expected to shift the NBE emission from interfacial regions; however, this effect may not be significant enough to affect bulk emission.^41^ Cu doping was too low and likely well dispersed to induce significant changes in the bulk electronic structure. Defect passivation can counterbalance band bending or carrier concentration effects that contribute to maintaining the NBE energy; however, it can have an impact on band intensity. Fewer surface defects per unit volume would reduce nonradiative recombination centers at the surface and thus increase NBE band intensity,^42^ which is expected upon increasing Cu loading. This is the case observed for CZO films with low Cu contents (CZO-2.2, CZO-3.7), as shown in Figure 4b, where the NBE band intensity increases. At the same time, large Cu NPs can act as recombination centers or contribute to interfacial defects, potentially decreasing the NBE band intensity (CZO-15.2). It is worth noting that the plasmonic coupling from Cu NPs can also impact the NBE band intensity, following a similar nonlinear correlation: too-small NPs result in poor field enhancement and thus weak SPR, while too-large NPs can lead to radiative damping and scattering, decreasing field enhancement again. The strongest NBE intensities recorded corresponded to samples CZO-3.7 and CZO-5.6 (Figure 4b). An enhanced NBE emission has been attributed to SPR upon injection of hot electrons from metal NPs into the conduction band of semiconductors (Figure 4c).^43,44^ Some authors have demonstrated an SPR effect in Cu NPs deposited on titania (TiO_2_) substrates after prolonged exposure to air oxidation.^45^ Charge transfer from Cu NPs into ZnO has also been demonstrated through an amorphous CuO layer, resulting in an enhancement of photocatalytic performance.^44,46^

The CZO films were assessed as photoanodes in a three-electrode electrochemical cell using a 0.5 M Na_2_SO_4_ electrolyte solution at pH 7.0. Figure 5 shows current–voltage (*J–V*) curves in the dark and under simulated sunlight conditions (AM = 1.5 G, 100 mW cm^–2^). The onset potential of these films shifted cathodically compared with that of pure ZnO, ranging from ∼0.55 *V*_RHE_ (CZO-0) to ∼0.35 V_RHE_ (CZO-15.2), indicating an enhanced performance of the Cu-modified photoanodes.^47^ The corresponding current densities are given in Table 1, following an almost linear trend with Cu loading. The best performance was obtained from sample CZO-5.6, reaching *ca*. 1.22 mA cm^–2^ (at 1.23 *V*_RHE_). This is in line with previous reports demonstrating enhanced photocatalytic properties upon incorporation of Cu into ZnO materials.^48,49^ The stability of the CZO-15.2 sample was compromised at high voltages, as inferred from an inspection of Figure 5.

**Figure 5:**
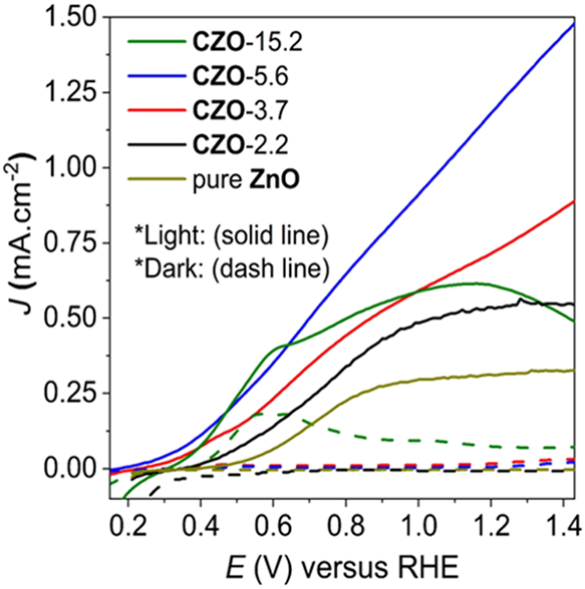
Photoelectrochemical properties of pure ZnO and CZO photoanodes providing *J–V* curves obtained in the dark and under AM 1.5 G illumination.

Further work explored strategies to improve the stability of the photoanodes, as they exhibited corrosion within 1 h under the conditions used in these experiments (0.1 *V*_Ag/AgCl_, AM 1.5 G). In particular, a thin amorphous TiO_2_ protective coating was deposited onto the best-performing photoanode (i.e., sample CZO-5.6) by using atomic layer deposition. An optimal thickness of 7 nm with respect to effective levels of protection and hole transfer was chosen using guidance from previous studies in the literature for PEC electrodes.^29,30^ The presence of TiO_2_ was confirmed by EDS analysis, and the dominant Ti 2p_3/2_ peak was identified at 458.4 eV in XPS analysis (Figure S2). XRD studies showed no indication of crystalline TiO_2_ phases after deposition. As shown in Figure 6a, the TiO_2_ layer contributed to the photocurrent density of the film, improving its performance to 1.43 mA cm^–2^ at 1.23 *V*_RHE_, with a photocurrent onset potential of ∼0.35 *V*_RHE_. This result is significantly higher than that of other Zn-based anodic materials reported in the literature (Table 2). The enhanced photocatalytic activity upon deposition of an amorphous TiO_2_ coating has been attributed to a facile transfer of holes across the semiconductor–electrolyte interface.^50^ This strategy also increased the stability of the photoanode by tenfold, with a useful half-life of ∼3 h (Figure 6b). The extent of corrosion after that time was evaluated by monitoring the Cu­(II) satellite peak in XPS (Figure S3), with the formation of CuO and a concomitant color change from green to dark yellow.

**Figure 6:**
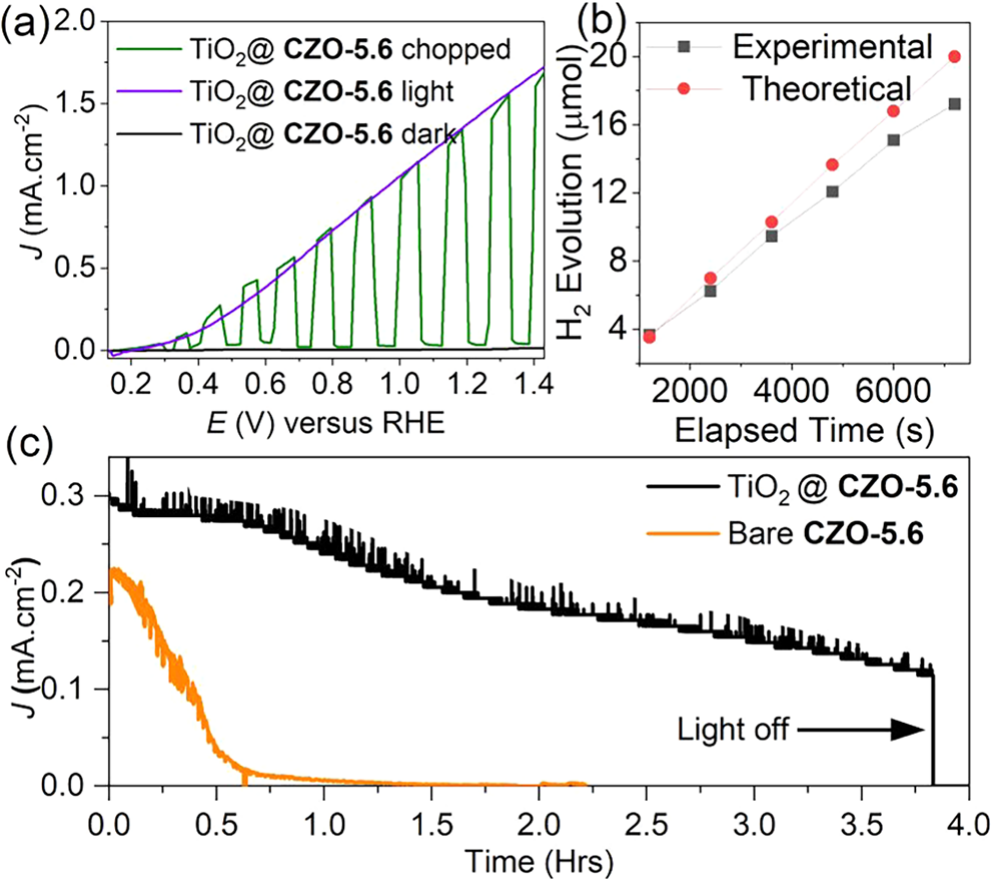
(a) Photocurrent density–voltage curves obtained from sample TiO_2_@CZO-5.6 under dark, light, and chopped AM 1.5 G illumination. (b) Corresponding experimental and theoretical H_2_ rates under continuous illumination (AM 1.5 G 100 mW·cm^–2^) with a bias voltage of 0.6 *V*_Ag/AgCl_. (c) Stability of bare CZO-5.6 and TiO_2_@CZO-5.6 samples measured with a bias of 0.5 *V*_RHE_ under AM 1.5 G sun illumination.

**Table 2:** Comparison of Photocurrent Densities (*J*, at 1.3 V) from Different ZnO-Based Electrodes Reported in the Literature and This Work^a^

photoelectrode	deposition method	*J* /mA cm^–2^	RE	refs
ZnO nanostructure	thermal oxidation, magnetron sputtering	0.05	Ag/AgCl	^51^
ZnO nanostructure	pulsed laser deposition	0.20	Ag/AgCl	^52^
ZnO plate film	electrodeposition	0.4	RHE	^53^
N-doped ZnO wire	hydrothermal deposition + annealing	0.5	Ag/AgCl	^54^
graphene-ZnO	alkali precipitation + solvothermal	0.6	Ag/AgCl	^55^
defect-rich ZnO rods	microwave-assisted chemical bath	0.705	RHE	^56^
ZnO nanostructure	solution processing + dip-coating	1.00	RHE	^57^
TiO_2_@Cu-ZnO film	chemical vapor deposition	1.43	RHE	this work

a It is important to note that the testing conditions (e.g., electrolyte composition, pH, and reference electrode) are not identical across all tests. Please see the references for further information.

The H_2_ generated from the optimized photoanode, TiO_2_@CZO-5.6, under a bias of 0.6 *V*_Ag/AgCl_ and AM 1.5 sun illumination, increased linearly and fitted the theoretical value, as shown in Figure 6c. The photoanode exhibited a high and stable Faradaic efficiency of 89% for the 45 min duration of the stability test, indicating that the surface conditions promoted carriers to participate in the water-splitting reaction.

## Conclusions

Cost-efficient, optimized Cu-modified ZnO films synthesized by using chemical vapor deposition methods were used as photoanodes for solar-to-hydrogen evolution. The incorporation of Cu nanoparticles enhanced the photocatalytic properties of the photoanode, which was mainly attributed to surface plasmon resonance (SPR) effects, based on absorbance and photoluminescence evidence. The addition of an amorphous TiO_2_ protective coating to the best-performing film significantly enhanced its photocurrent density above average reported in the literature. The protective coating contributed to a substantial extension (tenfold) of the useful life of the photoanode. This encouraging result represents a step forward in the fabrication of optimized photoanodes for green H_2_ generation, and it underscores the need for the implementation of synthesis strategies for high-surface-area photoanodes.

## Supplementary Material



## Data Availability

Data for this article are available upon request from the corresponding authors.

## Notes

Film growth setup, spectral irradiance of the AM 1.5 G standard solar spectrum, and additional material characterization (PDF)
