# Supplementary material for: Optimized Copper-Modified Zinc Oxide Photoanodes for Solar-to-Hydrogen Evolution
Source: ACS Appl Mater Interfaces. 2026 Jan 10;18(3):5061–8. doi: 10.1021/acsami.5c17721 (PMC12862751; doi:10.1021/acsami.5c17721)
Supplement: Supplementary file 1 [file am5c17721_si_001.pdf]

Supporting Information

Optimized Copper-Modified Zinc Oxide Photoanodes for Solar-to-Hydrogen Evolution

Premrudee Promdet,[a]* Fan Cui,[b] Raul Quesada-Cabrera,[a,c] Sanjayan Sathasivam,[a,d,e] Jiang Wu,[f] Claire J. Carmalt[a] and Ivan P. Parkin[a]*

(a) Materials Chemistry Centre, Department of Chemistry, UCL (University College London), 20 Gordon Street, London WC1H 0AJ, UK
(b) Department of Electronic & Electrical Engineering, UCL (University College London), Malet Place, London WC1E 7JE, UK
(c) Department of Chemistry, Institute of Environmental Studies and Natural Resources (i-UNAT), Universidad de Las Palmas de Gran Canaria (ULPGC), Campus de Tafira, Las Palmas 35017, Spain
(d) School of Engineering & Design, London South Bank University, 103 Borough Road, London, SE1 0AA, UK
(e) Energy, Materials & Environment Research Centre, London South Bank University, 103 Borough Road, London, SE1 0AA, UK
(f) Institute of Fundamental and Frontier Sciences, University of Electronic Science and Technology of China, Chengdu 610054, P. R. China

Email: premrudee.promdet.17@ucl.ac.uk & i.p.parkin@ucl.ac.uk

Supplementary Information

Experimental Setup

Cu:ZnO thin films were deposited by using aerosol-assisted chemical vapor deposition (AACVD), as detailed in the experimental section. Firstly, the mist of the precursor solution (zinc acetate dihydrate, copper acetate monohydrate and methanol) was generated by an ultrasonic humidifier. Then, the vaporized liquid mixture was transported to a tube furnace using nitrogen as carrier gas. The precursors react followed by the deposition of Cu:ZnO on the heated substrate. Meanwhile, the waste products and unreacted precursors were carried away to the exhaust.

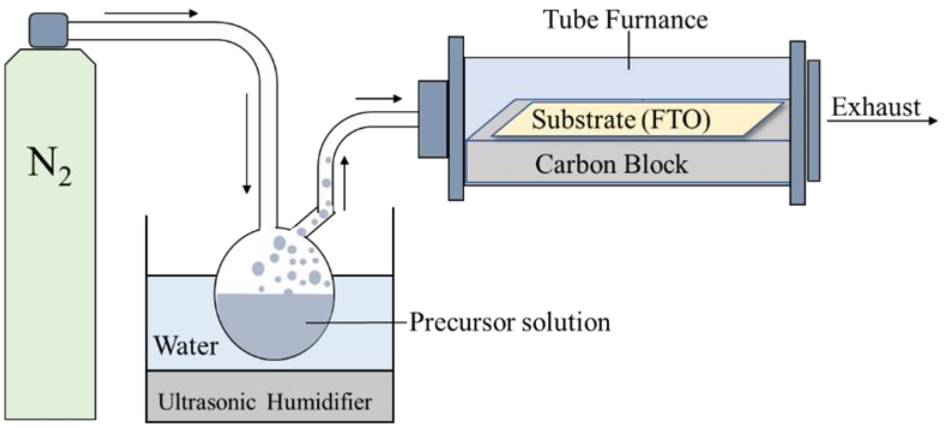

Scheme S1. The illustration of principle of AACVD operation used for the deposition.

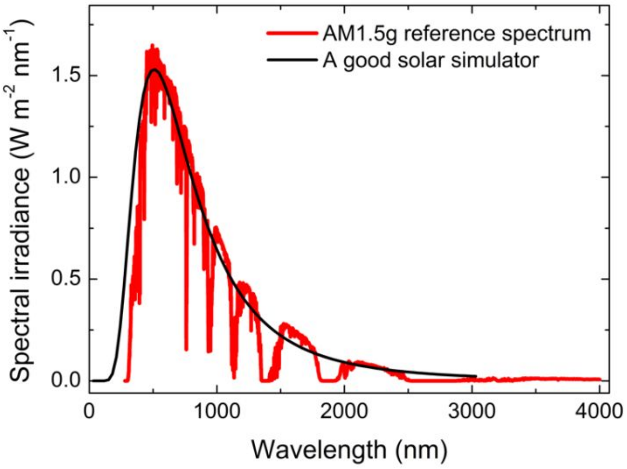

Fig. S1. Spectral irradiance of the AM 1.5G standard solar spectrum (red) and of an ideal solar simulator produced with a 5700 K blackbody irradiance spectrum (black)

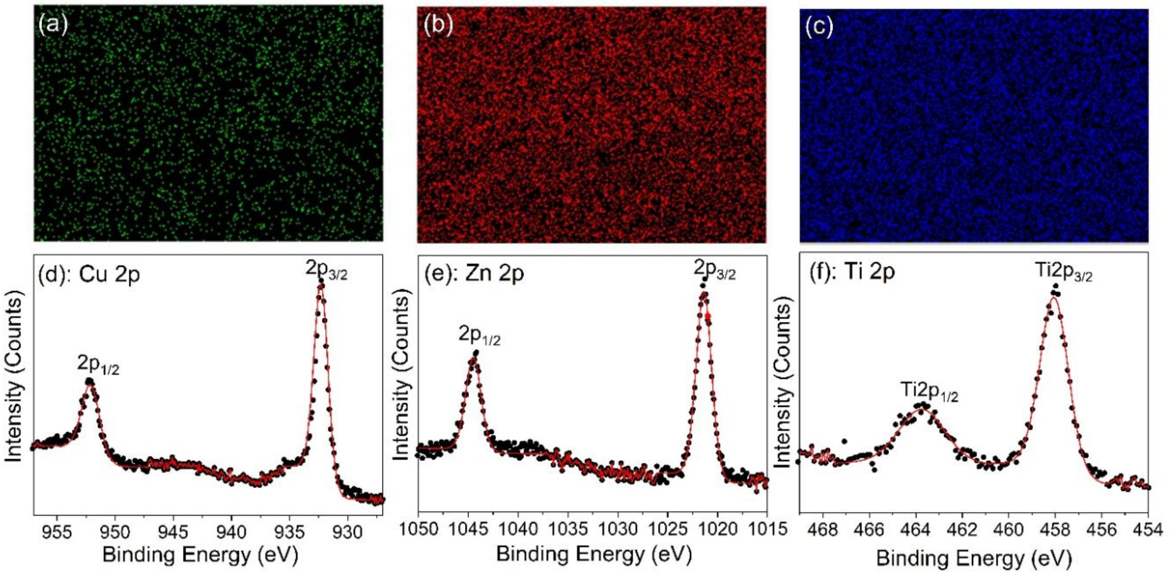

Fig. S2. EDS elemental mapping images showing the distribution of chemical elements; (a) Cu, (b) Zn and (c) Ti, and XPS showing chemical composition; (d): Cu, (e): Zn and (f): Ti, of CZO-5.6 coated with amorphous TiO_2 (TiO_2@ CZO-5.6).

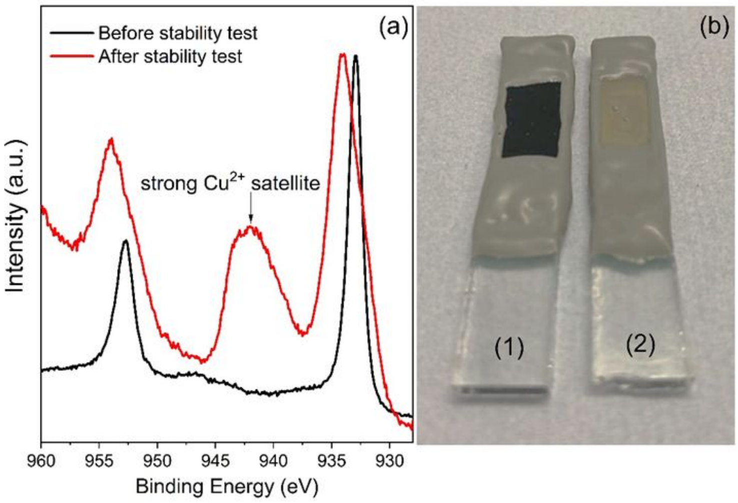

Fig. S3. (a) XPS spectra revealing Cu2p of TiO_2@CZO-5.6 film before and after stability test in 0.5 M Na_2SO_4 in a three-electrode electrochemical cell and (b) The pictures of TiO_2@CZO-5.6 (1) before and (2) after stability test measured in 0.5 M Na_2SO_4 in conditions of AM 1.5 G illumination. The colour change observed from black to yellow/green for the before and after stability electrodes is likely due to the formation of CuO or $Cu(OH)_2$ species.
